# BIRC3 is a novel driver of therapeutic resistance in Glioblastoma

**DOI:** 10.1038/srep21710

**Published:** 2016-02-18

**Authors:** Dapeng Wang, Anders Berglund, Rajappa S. Kenchappa, Peter A. Forsyth, James J. Mulé, Arnold B. Etame

**Affiliations:** 1 Department of Neuro-Oncology, Moffitt Cancer Center, 12902 Magnolia Drive, Tampa, FL 33612 USA; 2 Department of Tumor Biology, Moffitt Cancer Center, 12902 Magnolia Drive, Tampa, FL 33612 USA; 3 Department of Medical Bioinformatics, Moffitt Cancer Center, 12902 Magnolia Drive, Tampa, FL 33612 USA; 4 Department of Immunology, Moffitt Cancer Center, 12902 Magnolia Drive, Tampa, FL 33612 USA; 5 Department of Cutaneous Oncology, Moffitt Cancer Center, 12902 Magnolia Drive, Tampa, FL 33612 USA

## Abstract

Genome-wide analysis of glioblastoma (GBM) reveals pervasive aberrations in apoptotic signaling pathways that collectively contribute to therapeutic resistance. Inhibitors of apoptosis proteins (IAP) exert critical control on the terminal segment of apoptosis leading to apoptosis evasion. In this study, we uncover a unique role for BIRC3, as an IAP that is critical in GBM in response to therapy. Using the TCGA dataset of 524 unique samples, we identify BIRC3 is the only IAP whose differential expression is associated with long-term survival in GBM patients. Using patient tissue samples we further show that BIRC3 expression increases with recurrence. When extrapolated to a preclinical model of a human GBM cell line, we find an increase in BIRC3 expression in response to irradiation (RT) and temozolomide (TMZ) treatment. More importantly, we mechanistically implicate STAT3 and PI3K signaling pathways as drivers of RT-induced up-regulation of BIRC3 expression. Lastly, we demonstrate that both *in-vivo* and *in-vitro* BIRC3 up-regulation results in apoptosis evasion and therapeutic resistance in GBM. Collectively, our study identifies a novel translational and targetable role for BIRC3 expression as a predictor of aggressiveness and therapeutic resistance to TMZ and RT mediated by STAT3 and PI3K signaling in GBM.

Therapeutic resistance is a hallmark of glioblastoma multiforme (GBM) making disease recurrence inevitable. Despite advances in the multimodal strategies of surgical resection, radiotherapy (RT) and chemotherapy with Temozolomide (TMZ), the median survival for newly diagnosed patients hovers around 14 months^1^. Moreover, the prognosis is markedly dismal for patients with recurrent GBM, where median survival of 3–9 months with standard chemotherapy^2^,^3^ and 6-month progression-free survival rates of 15–16%^2^,^4^,^5^ are often realized. Evasion from apoptosis is central to cancers in general^6^, and GBM is no exception. Genome-wide analysis of GBM reveals pervasive aberrations in multiple apoptotic pathways^7^. For instance several critical anti-apoptotic signaling pathways such as the Epidermal Growth Factor Receptor (EGFR), Platelet Derived Growth Factor Receptor (PDGFR), Phosphatidylinositide 3-kinase [PI3K], and Signal Transducer and Activator of Transcription (STAT3) are highly activated in GBM^7^. Furthermore, aberrancies of the anti-apoptosis BCL-2 family^7^,^8^, mutations in apoptosis-related tumor suppressor proteins such as TP53^7^, and increase expression of Inhibitor of Apoptosis Proteins (IAP)^9^,^10^,^11^ collectively skew the apoptotic balance in GBM towards cell survival mechanisms, which all lead to therapeutic failure. Given the central role of anti-apoptosis signaling, strategies that define and target anti-apoptosis mechanisms could potentially ameliorate therapeutic resistance in GBM.

IAPs are characterized by the presence of baculoviral IAP repeat (BIR) domains^12^,^13^,^14^, highly up-regulated in GBMs^9^,^10^,^11^, and known to promote cellular survival in cancers through regulation of apoptosis^15^. Therefore, IAPs are emerging as attractive pharmacologic targets for ameliorating therapeutic resistance in cancers. Besides cell death^16^, IAPs also play a role in immunity and inflammation^17^. The human IAP family is composed of eight members: Neuronal IAP (NAIP), cellular IAP1 (c-IAP1) [BIRC2], cellular IAP2 (c-IAP2) [BIRC3], X-chromosome linked IAP (XIAP) [BIRC4], survivin [BIRC5], Apollon/Bruce [BIRC6)], Melanoma IAP (ML-IAP), and IAP-like Protein 2 (ILP-2)^16^. Only BIRC2, BIRC3, and BIRC4 regulate caspase activity^18^. BIRC4 directly inhibits caspases 3,7 and 9^19^,^20^,^21^,^22^,^23^,^24^, whereas the BIRC2 and BIRC3 proteins indirectly regulate caspase activation through E3 ligase activity, TNF-signaling and NFkB signaling^25^.

The central role of IAPs within the terminal segment of apoptosis has profound therapeutic and prognostic implications (Supplementary Figure 1). Since IAPs interact at the level of caspases, IAPs could serve as the definitive convergence point for signaling pathways that promote apoptosis evasion. Therefore, identifying and targeting critical IAPs that contribute to apoptotic evasion in GBM is a very rationale strategy. Higher expressions of IAP’s have been documented in malignant gliomas and often correlated with poor prognosis^9^,^10^,^11^. There is also preclinical evidence that targeting IAPs with small molecule inhibitors can reverse therapeutic resistance in GBM^26^,^27^. However, no studies to date have characterized the mechanistic impact of IAPs on therapeutic resistance and also on long-term survival in GBM. We therefore sought to understand the role of IAP expression on survival in a large cohort of GBM patients. We were interested in the role of IAP in the current standard GBM therapy of TMZ and RT. Detailed understanding of such mechanisms could permit optimized synergy between IAP targeting and standard therapy. Such a targeting strategy of downstream convergence signaling nodes could potentially overcome the current shortcomings of targeted GBM therapies that focus on upstream pathways.

The Cancer Genome Atlas (TCGA) provides a unique opportunity to examine GBM on a larger scale both clinically and molecularly since TCGA contains expression data from over 527 unique GBM samples^7^. Using TCGA data in this study, we identified BIRC3 as a critical determinant of survival in GBM patients. BIRC3 was the only IAP among several IAPs whose differential expression was significantly related to the 5-year survival in patients with GBM. Lower expression levels of BIRC3 were associated with a markedly favorable outcome. Given the above observations, we sought to further delineate the unique mechanistic role of BIRC3 in GBM therapeutic resistance in this study. For the first time, we demonstrate that BIRC3 expression increases secondary to acquisition of TMZ and RT resistance. In addition, BIRC3 emerges as a novel driver of therapeutic resistance in GBM. Furthermore, for the first time, we mechanistically implicate BIRC expression as a downstream signaling node for STAT3 and PI3K in response to GBM therapy. BIRC3 therefore emerges as a rational target with translational implications.

## Materials and Methods

### Mice

6–8 weeks female NCRNU athymic mice were ordered from Taconic Biosciences. All animals were housed in the American Association for Laboratory Animal Care–accredited Animal Resource Center at Moffitt Cancer Center. Experiments were all carried out under protocols approved by the Institutional Animal Care and Use Committee.

### Cancer Genome Atlas (TCGA) analysis of BIRC3 expression in GBM patients

The TCGA data portal was used to download clinical, UNC IlluminaHiSeq_RNASeqV2 Level 3 and BI HT_HG-U133A Level 1 data (Jun-2015) for GBM. For the RNAseq data the rsem.genes.normalized_results files (n = 169) were used without further normalization. The RNAseq data were grouped into Primary Solid Tumor (n = 156) and Recurrent Solid Tumor (n = 13) based on the TCGA annotation. The Affymetrix U133A CEL files (n = 548) were normalized with IRON^28^ using sample 5500024037497121008340.A12.CEL as median sample. The CEL file with the smallest RMSD was selected if there were multiple CEL files for a single sample, resulting in 529 files, 524 of which had available survival data.

The Affymetrix U133 Plus 2.0 CEL files (n = 284) for GSE16011 were downloaded from GEO and normalized with IRON^28^ using the GSM405254.CEL as the median sample. Five samples (GSM405240, GSM405364, GSM405370, GSM405414, and GSM405468) were removed since their expression of XIST and DDX3Y did not match their gender from clinical data. In the survival analysis, only samples classified as GBM (n = 157) and with survival data (n = 153) were used.

The median cut was used in all survival analysis and log rank p-value was calculated. Group comparisons were done using Mann-Whitney test and the two-tailed p-value is reported. QC and PCA were done using Evince (www.umbio.com) and MATLAB version R2014b. Survival analysis was performed in GraphPad Prism version 6.0d for Mac OS X, GraphPad Software (La Jolla, CA, USA).

### Cell lines and reagents

U87 and U251 human glioblastoma cell lines were cultured in DMEM (life technologies, USA) supplemented with 10% fetal bovine serum (Sigma-Aldrich), 100 units/ml penicillin-100 μg/ml streptomycin (Sigma-Aldrich). The culture was maintained at 37 °C in a humidified atmosphere containing 5% CO_2_. Temozolomide (TMZ) and Ly294002 were obtained from Sigma-Aldrich. S3I-1757 was kindly provided by Dr. Said Sebti (Moffitt Cancer Center). PIK-75, TGX221, IC-87114 and AS-605240 were purchased from Cayman Chemical (USA). G418 sulfate (50 mg/ml) was obtained from Life Technologies (USA). Stat-3 siRNA was obtained from Sigma-Aldrich (USA). Rabbit anti-Human-Akt, Rabbit anti-Human phospho-Akt, Goat anti-Rabbit IgG-HRP, anti- β-actin IgG-HRP and Rabbit anti-Human cIAP2 were obtained from Santa Cruz Biotech (USA).

### Development of BIRC3 over expressing U251 cell

Human BIRC3 expression construct (Bacterial stock) was obtained from Genecopoeia (USA). A single bacteria clone was picked from a freshly streaked LB plate containing100 μg/ml ampicillin and inoculated to a culture of 3 ml LB medium containing 100 μg/ml ampicillin, which was then incubated for 12–16 hours at 37 °C with vigorous shaking. Plasmid was purified using QuickLyse Miniprep Kit (Qiagen, USA). U251 (2 × 10^5^) was seeded in 400 μL culture medium in 6 well tissue culture plate 24 hours before transfection. BIRC3 expression plasmid (4 μg) was diluted in 400 μL per well of serum-free DMEM. TurboFect Transfection Reagent (6 μL; Thermo Scientific, USA) was added to the diluted plasmid, votexed and incubated for 20 minutes at room temperature. Plasmid-transfection reagent mixture (400 μL) was added drop-wise to each well of U251. The plate was gently rocked to achieve even distribution of the complexes, and then incubated at 37 °C in a CO_2_ incubator for 6 hours and replaced with fresh culture medium. G418 sulfate (500 μg/ml) was used for selection 48 hours after transfection and the G418 sulfate concentration was then reduced to 200 μg/ml 7 days later for maintenance. The over expression of BIRC3 was verified by Western-blot.

### Development of TMZ-resistant U87 Cell

To establish the TMZ resistant cell line, U87 cells were exposed to stepwise increasing concentrations of TMZ (up to 50 μM) over a period of 4 months as described previously^29^. In short, U 87 cells were cultured in the presence of 3.125 μM TMZ. The concentration was then increased by 2-fold for every two passages until it reached 50 μM. After 2 months, selected TMZ resistant U87 cells were used for the downstream experiments.

### X-ray irradiation

5 × 10^5^ U87 cells were seeded in a T25 flask 24 hours prior irradiation (RT). The next day, U87 cells were irradiated using XRAD 160(Precision X-ray Inc, USA) at the rate of 2.5 Gy/minute.

### Real-time PCR

Total RNA was extracted using TRIzol (Life technologies, USA). RNA was quantified with Nanodrop 2000 (Thermo Scientific, USA). cDNA was synthesized using 1 μg total RNA and the iScript cDNA Synthesis Kit (Bio-Rad, USA). BIRC3 real-time PCR was performed using the Bio-Rad CFX96 Touch Real-Time PCR Detection system with human BIRC3 forward primer: AAGCTACCTCTCAGCCTACTTT; and the reverse primer: CCACTGTTTTCTGTACCCGGA. β-actin was used as the internal control. The sequences of β -actin PCR primers were as follows: forward primer: TCCTGTGGCATCCACGAAACT and reverse primer: GAAGCATTTGCGGTGGACGAT. The PCR program was: 95 °C 10 min, 1 cycle; 95 °C 15 s −>60 °C 30 s −>72 °C 30 s, 40 cycles; 72 °C 10 min, 1 cycle.

### siRNA transfection

U87 cells were transfected with STAT3 small interfering RNA (siRNA; 80 nM, Sigma-Aldrich) or control siRNA (80 nM) using Lipofectamine RNAiMAX (Life Technologies, Carlsbad, CA, USA). Briefly, one day prior to transfection, the cells were cultured in T25 flasks (5 × 10^5^) with 10% FBS DMEM without antibiotics. An siRNA-lipofectamine complex mixture in serum-free Opti-MEM (Life Sciences, USA) was prepared according to the manufacturer’s instructions and was added to the cells. The medium was replaced with DMEM containing 10% FBS 5 hr after transfection, and the cells were then exposed to X-ray irradiation 48 hours after transfection prior to analysis.

### Western-blot analysis

Forty μg of heat-denatured proteins were loaded on 4–15% precast polyacrylamide gel (Bio-Rad, USA). The proteins were then transferred to PVDF membranes (Bio-Rad, USA.), which were blocked with 5% non-fat milk solutions for 1 hour at room temperature. The target proteins were then detected by the primary antibody at 4 °C overnight, washed with 0.1% Tween-PBS and incubated with appropriate secondary antibody for 2 hours. The membranes were then washed and the target proteins were detected with luminol reagent and X-ray film (Santa Cruz). Quantification of the target protein was done using Photoshop (Adobe,USA). In short, the background of the target protein and β-actin were subtracted. Then, the relative expression of the target protein was normalized to β-actin and compared to that of the control group in each experiment.

### Survival assay

Survival of U251 cells was measured using an XTT Cell Viability Assay Kit (Cell Signaling). In short, following treatment/transfection of U251 cells, 50 μL of XTT detection solution was added to each well of a 96-well plate (containing 200 μL/well of culture medium) and incubated at 37 ^o^C for 1 hour. The absorbance was then measured at 450 nm using a microplate reader (Molecular Device, USA). The relative survival was calculated by dividing the absorbance of experimental group with that of the control group.

### Clone forming assay

Different numbers (100–2000) of U251 cells were seeded in a 100 mm dish and were treated with X-ray or TMZ the next day. The cells were then cultured until day 16. The culture medium was removed and cell clones were stained with crystal violet (EMD, USA) for 30 minutes. The dishes were gently washed with water and dried at room temperature overnight. Cell clones were then counted under a light microscope. A cell clone was defined to include at least 50 cells. The plating efficiency (PE) was calculated by:

PE = 100 × number of clones counted/number of cells plated. The survival fraction (SF) was calculated by: SF = 100 × PE of treated group/PE of control group.

### Caspase3/7 activation assay

Caspase3/7 activation was detected using the Cell Event Caspase-3/7 Green Flow Cytometry Assay Kit as described by the manufacturer (Life Sciences, USA). In brief, flow cytometry tubes were prepared with each containing 1 ml of cell suspension at 1 × 10^5^ −1 × 10^7 ^cells/ml; 1 μL of Cell Event Caspase-3/7 Green Detection Reagent was then added to each sample and incubated for 25 minutes at 37 °C. The samples were read on a BD LSRII flow cytometer and analyzed by FlowJo software (Tree Star, USA).

### U251 flank model and immunohistochemistry

Two million U251 or U251 cIAP2 over-expressing cells were injected in the flank of female NCRNU athymic mice. Two weeks later, mice were subjected to TMZ treatment (5 mg/ml, oral, daily). Tumor samples were taken after 5 doses of TMZ treatment, and were fixed with 10% neutral-formalin buffer for 24 hours. The samples were then dehydrated, paraffin-embedded and sectioned. Sections were dewaxed, treated with 3% H_2_O_2_ for 10 min and incubated with anti-cleaved Caspase 3 antibody (1:100 dilutions) overnight at 4 °C. Biotinylated secondary antibody (1:200 dilutions) was added at room temperature for 1 hour, followed by the incubation with ABC-peroxidase for additional 1 hour. After washing with Tris-buffer, the sections were incubated with DAB (3, 30 diaminobenzidine, 30 mg dissolved in 100 ml Tris-buffer containing 0.03% H_2_O_2_) for 5 min, rinsed in water and counterstained with hematoxylin.

### U251 apoptosis detection by live animal imaging

The U251 flank model was set up as described above. The level of U251 apoptosis after TMZ treatment was monitored *in vivo* using FMT-2500LX (PekinElimer, USA) and NIR-FLIVO 747 *in vivo* Apoptosis Kit (ImmunoChemistry Technologies, USA). Fifty μL 1X NIR-FLIVO were injected into the lateral tail vein of the treatment and control (untreated) animals. NIR-FLIVO was allowed to circulate within the mice for 4-hours for optimal image acquisition. The animals were then anesthetized and imaged using FMT-2500LX. The exciting and emission wavelength was 747 nm and 776 nm, respectively.

### Statistics

Student’s t-test was used for all the studies unless indicated. P < 0.05 was considered significant difference. *means P < 0.05.

## Results

### BIRC3 Gene Expression Correlates Inversely with Outcomes in GBM Patients

**Figure 1:**
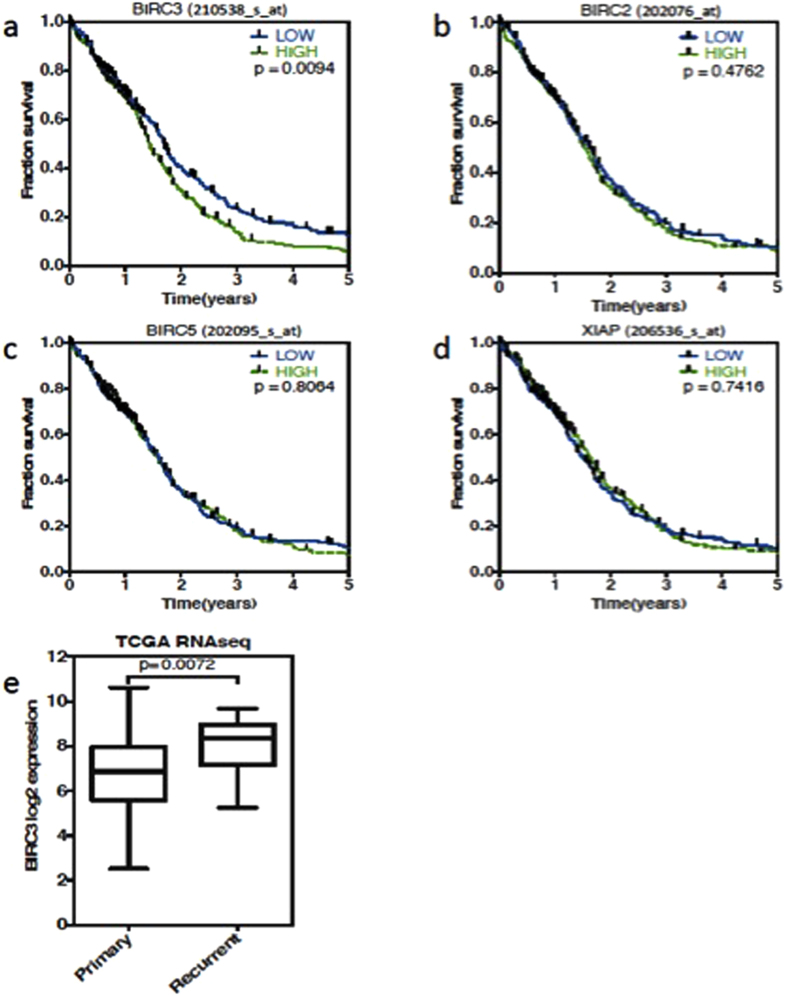
The Cancer Genome Atlas (TCGA) shows BIRC3 expression is related to the survival of GBM patients. The relationship of IAPs expression with GBM patient survival. 524 unique GBM samples on the Affymetrix U133A chip were analyzed. (**a**) BIRC3(c-IAP2) is the only IAP family member which is related to 5-years overall survival in TCGA GBM data (p < 0.01). (**b**) BIRC2 (c-IAP1). (**c**) BIRC5 (Survivin). (**d**) BIRC4 (XIAP). Results are shown using median cut. (**e**) BIRC3 was expressed at a higher level in recurrent GBM patients (n = 13) than that of newly diagnosed GBM patients based on boxplot (n = 156).

To assess the potential clinical impact of IAPs in GBMs, we examined gene expression data using The Cancer Genome Atlas (TCGA) microarray expression dataset from 524 unique GBM samples. This dataset has both molecular and clinical information. When we examined the association of IAP expression on overall survival (OS) of GBM, BIRC3 was the only IAP whose differential expression was related to the 5-year OS in GBM. Patients with down-regulated expressions of BIRC3 demonstrated a statistically significantly enhanced OS compared to patients with higher BIRC3 expression (Fig. 1a) Of note, differential expressions of BIRC5 (survivin) and BIRC4 (XIAP) had no impact on 5-year OS in GBM patients (Fig. 1b–d) despite prior reports of a prognostic role based on tissue expression^9^,^11^. Thus, the TCGA data interrogation showed that BIRC3 is significantly associated with long-term OS of GBM patients (p < 0.01). To validate this finding in an independent data set, we investigated the effect of BIRC3 expression using another public GBM dataset, GSE16011, with 153 unique GBM samples. In this dataset, both the probe sets for BIRC3 confirmed the finding in the TCGA dataset (p = 0.025 & p = 0.022), (Supplementary Figure 2a - the result of only one probe set was shown here). Differential expressions of BIRC2, BIRC5 (survivin) and BIRC4 (XIAP) had no significant impact on OS in GBM patients in the GSE dataset as well using their respective probes (Supplementary Figure 2b–d). We also compared BIRC3 gene expression in newly diagnosed versus recurrent GBMs using the TCGA. Since the TCGA microarray set has data consisting solely of newly diagnosed patients, we instead employed the RNAseq expression data set that contains recurrent GBMs. Although the number of recurrent GBMs in was much smaller, BIRC3 was expressed at significantly higher level (p = 0.0072, group- median difference =1.5 log_2_ units) in recurrent GBM patients (n = 13) than that of newly diagnosed GBM patients (n = 156), see Fig. 1e. This finding raised the possibility that prior GBM therapy might up-regulate BIRC3 expression.

### TMZ Resistance and RT Promote Up-regulation of BIRC3 Gene Expression in GBM

**Figure 2:**
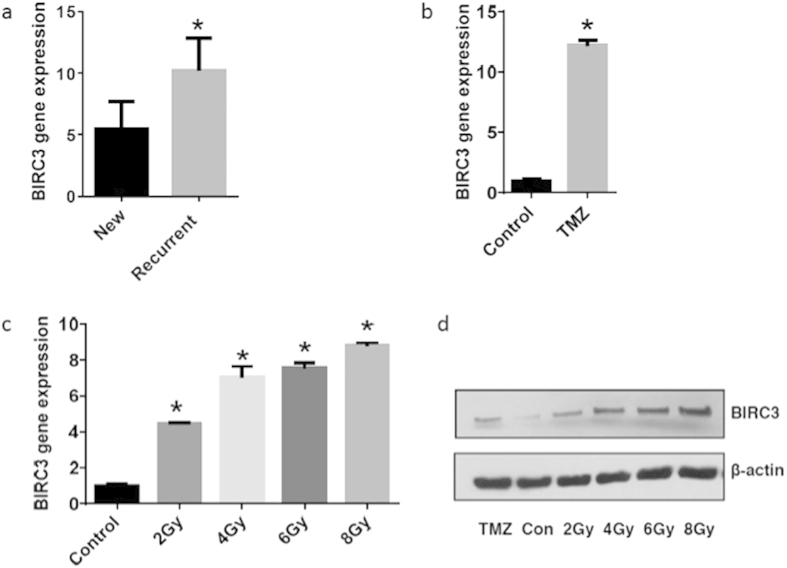
BIRC3 is up-regulated in therapy resistant GBMs. (**a**) BIRC3 gene is expressed at higher level in recurrent GBMs than in newly diagnosed GBMs. GBM samples were collected from newly diagnosed and recurrent GBM patients. Total RNA was extracted and cDNA was synthesized. BIRC3 gene expression was measured by RT-PCR analysis. (n = 7–8, p < 0.05). (**b**) TMZ drug resistant U87 cells were developed with gradual increase in TMZ concentration. Cells were harvested and total RNA was extracted and cDNA was synthesized. BIRC3 gene expression was measured by RT-PCR between WT-U87 and 50 μM TMZ resistant U87. (n = 3 independent experiments, p < 0.0001). (**c**) U87 cells were irradiated with increasing doses of X-ray. Cells were harvested and total RNA was extracted and cDNA was synthesized. BIRC3 gene expression was measured 24 hours later by RT-PCR. (n = 3 independent experiments, p < 0.05). (**d**) BIRC3 protein expression was increased upon TMZ treatment or X-ray irradiation in U87 cells. X-ray irradiated cells were harvested 24 hours after irradiation and Western blot analysis was performed using BIRC3 and actin antibodies. Data are representative of three independent experiments. p < 0.05.

Given the prognostic impact of BIRC3 within the TCGA dataset, we sought to examine its expression and association with GBM therapy using patient GBM tissue samples. In order to do so, we examined BIRC3 gene expression levels by RT-PCR in a subset of patients treated with RT +/− TMZ versus newly diagnosed non-treated GBM patients. Indeed, we observed a significantly higher expression of BIRC3 levels in previously treated GBMs patients compared to newly diagnosed GBM patients (Fig. 2a; P < 0.05). Thus, it appeared that BIRC3 expression increases with disease recurrence following standard GBM therapy.

To further determine the effects of GBM therapy (TMZ & RT) on BIRC3 expression, we examined human GBM cells that were rendered resistant to TMZ through step-wise exposures with two fold increases in TMZ concentration as well as GBM cell lines exposed to RT (see Materials and Methods). When U87 human GBM cell lines were rendered resistant to TMZ, we noted significant increases in BIRC3 expression as confirmed by RT-PCR, which correlated with acquisition of TMZ resistance (Fig. 2b; P < 0.0001). Next, in order to determine if RT treatment was also associated with BIRC3 expression up-regulation, we irradiated U87 GBM cells with 2–8 Gy. We noted a dose-dependent increase in BIRC3 expression with irradiation (Fig. 2c). Next we performed Western-blot which confirmed an increase in BIRC3 protein levels following TMZ and irradiation treatment (Fig. 2d). Collectively, these findings suggest that BIRC3 expression is induced as a consequence of standard GBM therapy and might serve as a prognostic marker for therapeutic resistance in GBM.

### Pharmacologic Inhibition of PI3K Signaling Blocks RT-induced Up-regulation of BIRC3 Expression in GBM

**Figure 3:**
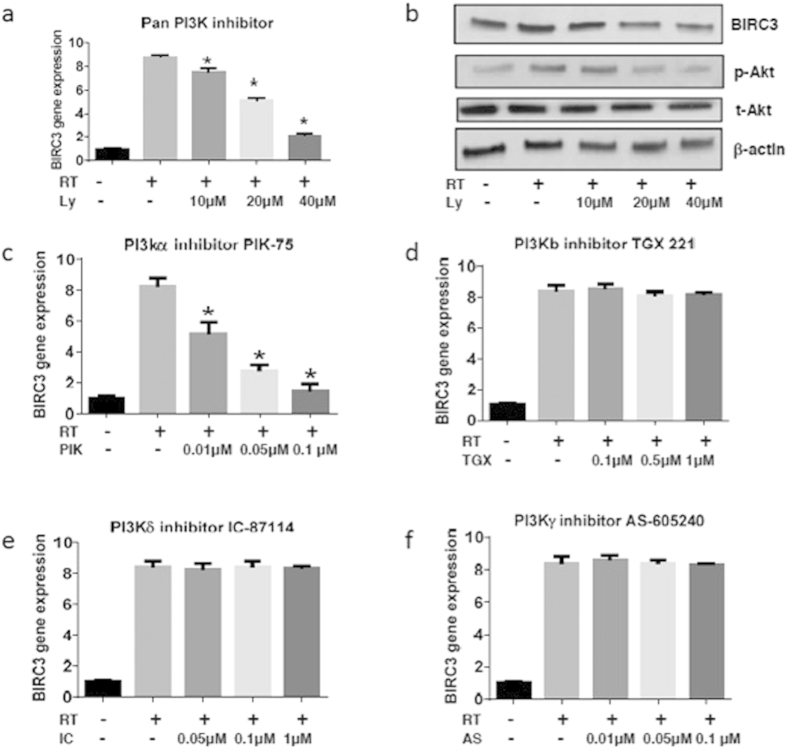
Up-regulation of BIRC3 in response to irradiation requires the PI3K pathway. (**a**) U87 cells were cultured in T25 flasks, pretreated with Ly294002 for 1 hour before X-ray exposure and were then irradiated. Total RNA was extracted and cDNA was synthesized in 24 hours. BIRC3 gene expression was analyzed by RT-PCR. (n = 3 independent experiments, p < 0.01). (**b**) U87 cells were pretreated with Ly294002 for 1 hour before X-ray exposure and then irradiated. BIRC3, phosphor-Akt and total Akt were analyzed by Western-blot 24 hours after X-ray irradiation. Data are representative of three independent experiments. p < 0.05. (**c–f**) U87 cells were pretreated with PI3K isoform inhibitors for 1 hour and then irradiated at 8 Gy. Cells were harvested 24 hours later and total RNA was extracted and cDNA was synthesized. BIRC3 gene expression was analyzed by RT-PCR. Similar results were obtained from three independent experiments. (p < 0.01 or no significant difference).

Since BIRC3 expression increased in response to standard therapy for GBM (TMZ/RT), we wanted to determine if this process was driven by other established upstream molecular pathway surrogates for therapeutic resistance in GBM. PI3K is highly activated in GBM and plays a major role in therapeutic resistance^7^,^30^. In addition, there is strong evidence that PI3K mediates apoptosis resistance in lung tumors^31^ and in endothelial cells^32^ through up-regulation of BIRC3. We therefore hypothesized that inhibition of PI3K could attenuate RT-induced up-regulation of BIRC3 in GBM cells. Indeed, pretreatment of U87 human GBM cell lines with a PI3K pan-inhibitor, LY294002 prior to irradiation with 4 Gy resulted in dose-dependent blockade of irradiation-induced up-regulation of BIRC3 (c-IAP2) expression by RT-PCR (Fig. 3a; P < 0.01). Western-blot confirmed reduction in BIRC3 and phospho-AKT protein levels when irradiated GBM cells were treated LY294002 (Fig. 3b). LY294002 is a pan-inhibitor of PI3K and therefore blocks all isoforms of PI3K.

To further characterize the role of PI3K, we evaluated several isoform-specific inhibitors. The alpha-isoform inhibitor, PIK-75, successfully attenuated irradiation-induced up-regulation of BIRC3 (c-IAP2) expression (Fig. 3c; P < 0.01). In contrast, inhibition of the beta-isoform with TGX 221, delta-isoform with IC87114, and gamma-isoform with AS605240 did not have any effect on irradiation-induced up-regulation of BIRC3 (c-IAP2) expression (Fig. 3d–f). These results suggest that PI3K signaling during irradiation contributes to BIRC3 (c-IAP2) up-regulation, which is reversed with PI3K inhibition. More specifically, the alpha isoform of PI3K appears to mediate this phenomenon. Therefore, BIRC3 serves as a downstream convergence node to PI3K signaling in response to RT in GBM.

### Inhibition of STAT3 Signaling Blocks RT-induced Up-regulation of BIRC3 Expression in GBM

**Figure 4:**
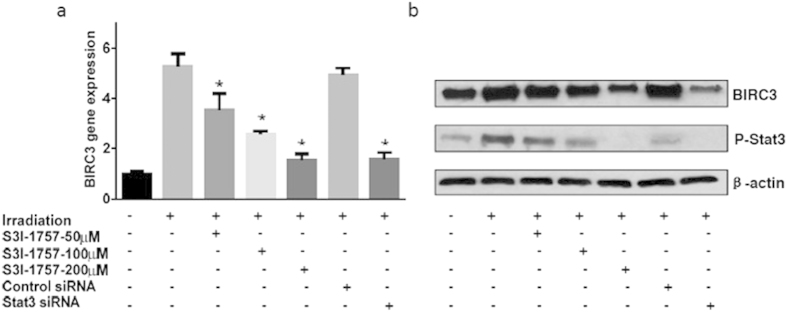
Up-regulation of BIRC3 in response to RT is dependent on the STAT3 Pathway. **(a**) U87 cells were cultured in T25 flasks and pretreated with the Stat3 inhibitor, S3I-1757 (1 hour earlier), transfected with Stat3 siRNA or control siRNA (2 days earlier) before X-ray irradiation. Total RNA was extracted and cDNA was synthesized in 24 hours. BIRC3 expression was analyzed by RT-PCR. (n = 3 independent experiments, p < 0.05). (**b**) U87 cells were treated as the same condition of experiment (**a**) and total protein was extracted 24 hours after irradiation. BIRC3 and p-Stat3 were analyzed by Western-blotting. Similar results were obtained from three independent experiments. p < 0.05.

STAT3 is another upstream signaling pathway that is implicated in therapeutic resistance in cancers^33^,^34^,^35^,^36^, and is highly activated in GBM^37^,^38^,^39^. Furthermore, there is strong mechanistic evidence that STAT3 mediates apoptosis resistance in intestinal cells through transcriptional activation of BIRC3^40^. We therefore hypothesized that inhibition of STAT3 could attenuate RT-induced up-regulation of BIRC3. Using a selective STAT3 homodimerization inhibitor S3I-1757^41^, we observed a dose-dependent blockade of irradiation-induced up-regulation of BIRC3 expression by RT-PCR (Fig. 4a; P < 0.05). Similar observations were made using siRNA where pretreatment with STAT3 targeting siRNA significantly blocked irradiation-induced up-regulation of BIRC3 (Fig. 4a; P < 0.05). STAT3-targeting siRNA produced similar levels of inhibition as 200 μM of S3I-1757. On the other hand, control non-targeting siRNA had no significant effect on irradiation-induced up-regulation of BIRC3. Western blot for phospo-STAT3 confirmed decreased activation of STAT3 with inhibitors (Fig. 4b). These results suggest that STAT3 signaling during irradiation contributes to BIRC3 up-regulation, which is reversed with STAT3 inhibition. Therefore, BIRC3 serves as a downstream convergence node to STAT3 signaling in response to RT in GBM.

### BIRC3 Expression Contributes to TMZ and RT Resistance in U251 Human GBM Cell Lines

**Figure 5:**
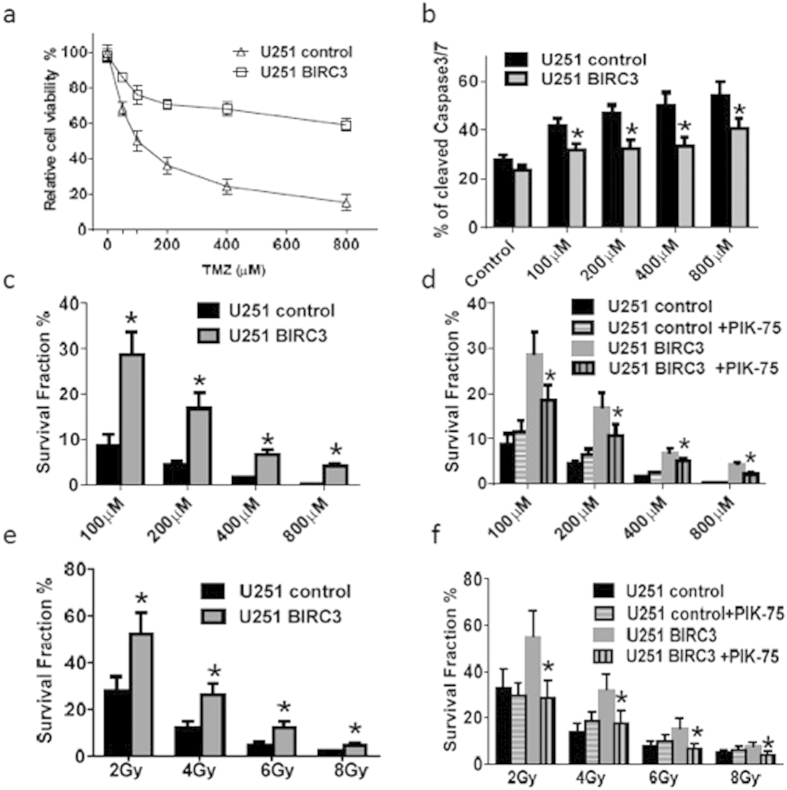
Over-expression of BIRC3 leads to TMZ and irradiation resistance in U251 cells via PI3Kα. (**a**) 0.2 × 10^4^ wild type U251 cells (U251 control) or BIRC3 over-expressing U251 cells (U251 BIRC3) were seeded in 96 well plates. Cells were then treated with TMZ (100–800 μM) for 5 days and cell survival was measured by the XTT Cell Viability Assay. The relative survival is shown. (**b**) 1–2 × 10^4^ U251 control or U251 BIRC3 cells were seeded in 24 well plates. Cells were then treated with 100–800 μM of TMZ for 5 days and caspase 3/7 activation was measured by a Cell Event Caspase-3/7 Green Flow Cytometry Assay Kit. (**c**,**d**) The clone forming ability (Survival fraction) of U251 control and U251 BIRC3 cells after treatment of TMZ (100–800 μM) were compared in the presence or absence of 0.1 μM PI3Kα specific inhibitor PIK-75. PIK-75 was applied 1 hour before TMZ treatment. (**e,f**) The clone forming ability (survival fraction) of U251 control and U251 BIRC3 cells after X-ray irradiation (2–8 Gy) were compared in the presence or absence of 0.1 μM PI3Kα specific inhibitor PIK-75. PIK-75 was applied 1 hour before irradiation. All experiments were repeated 3 times and one typical experiment is shown here. (n = 3 independent experiments, p < 0.05).

In an effort to further understand the BIRC3 phenotype and its effects on TMZ and RT treatment sensitivity, we generated U251 human GBM (U251 HG) cells with stable over-expression of BIRC3 confirmed by Western Blotting (Supplementary Figure 3). We then examined the relative cell viability as well as colony formation following either TMZ treatment or irradiation. When compared to control U251 HG cells, the BIRC3 over-expression U251 HG cells significantly enhanced viability to TMZ concentrations from 200 μM to 800 μM with more than 60% viability when compared to parental U251 HG cells (Fig. 5a; P < 0.05). Caspase cleavage was markedly diminished in BIRC3 over-expressing U251 HG cells, suggesting less apoptosis activity compared to control U251 in response to TMZ exposure (Fig. 5b; P < 0.05; Supplementary Figure 4). A similar trend was noted with colony formation where a statistically significant fraction of BIRC3 over-expressing U251 HG cells demonstrated an enhanced ability to form colonies compared to control U251 cells when exposed to either TMZ treatment (Fig. 5c; P < 0.05), or RT (Fig. 5e; P <  0.05). Interestingly, when both control U251 and BIRC3 over-expressing U251 HG cells were pretreated with a P1(3)K-alpha inhibitor (PIK-75) prior to exposure to varying concentrations of TMZ (Fig. 5d; P < 0.05) or RT (Fig. 5f; P < 0.05), there was a significant inhibition of colony formation even for the over-expression construct. This was somewhat surprising since that would imply that PIK-75 inhibited exogenous BIRC3 as well. PIK-75 effect was not pronounced in the absence of BIRC3 over expression probably due to very low levels of endogenous BIRC3 in U251 HG cells at baseline. It is therefore likely that PI3K blockade might oversensitive GBM cells in the presence of BIRC3 up-regulation. Taken together, the TMZ and RT data suggest that the BIRC3 phenotype contributes to therapeutic resistance in human GBM cells. Furthermore, PI3K-alpha inhibition can block BIRC3-mediated therapeutic resistance to RT and TMZ treatment in GBM.

### BIRC3 expression inhibits apoptosis in TMZ-treated human xenografts

**Figure 6:**
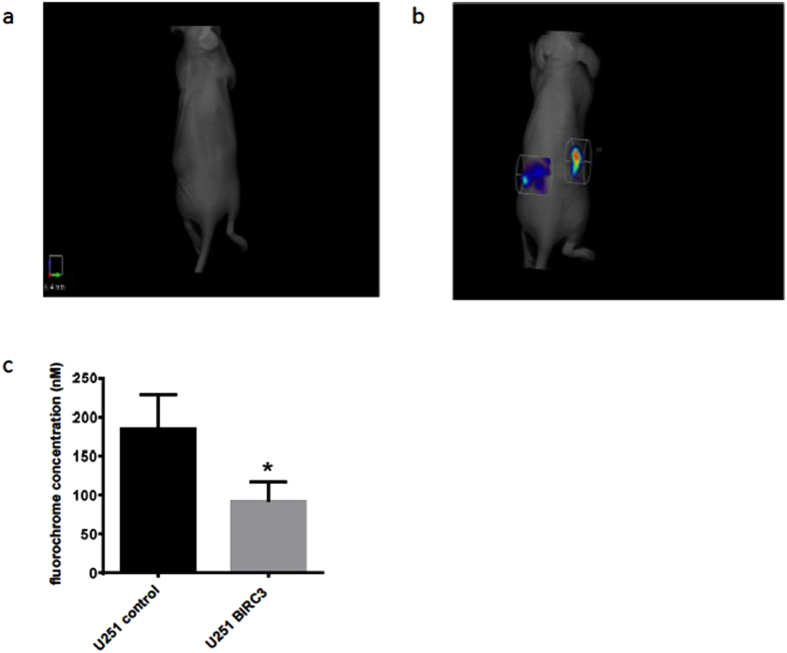
BIRC3 expression (n = 3 independent experiments, p < 0.05) treatment induced apoptosis *in vivo*. Mouse GBM xenografts were set up by injecting 2 × 10^6^ U251 control or U251 BIRC3 cells on the right and left flank, respectively, of the same mice. Two weeks later, mice with similar tumor size on both sides were treated with either 50 mg/kg of TMZ orally or vehicle control daily for 5 days. Mice were injected with 50 μL NIR-FLIVO747 via the lateral tail vein after the 5th dose of TMZ. 4 hours later, mice were imaged using the PerkinElmer FMT2500 to compare tumor cell apoptosis on both sides. U251 control cells were injected in the right flank while U251 BIRC3 cells were injected in the left flank. (**a**) Vehicle treatment. (**b**) TMZ treatment. Data are representative of 5 mice. (**c**) Average fluorochrome concentration in the tumor area on both sides (p < 0.05; n = 5).

**Figure 7:**
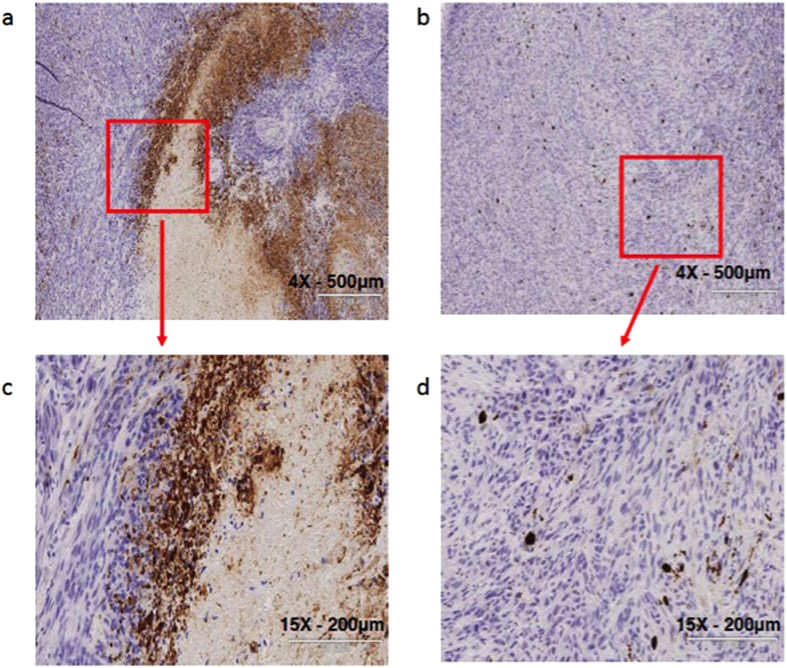
Decreased apoptosis in TMZ treated BIRC3 over-expressing xenografts. Mouse GBM xenografts were set up by injecting 2 × 10^6^ U251 control or U251 BIRC3 cells on the right and left flank, respectively, of the same mice. Two weeks later, mice with similar tumor size on both sides were treated with either 50 mg/kg of TMZ orally or vehicle control daily for 5 days. Mice were then sacrificed and tumor tissue on both sides were isolated, cut and fixed in 10% neutral formalin. Cleaved caspase3 immunohistochemistry was performed as described in the Material and Methods section. (**a,c**) U251 control. (**b,d**) U251 BIRC3. 5 mice were included in this study and similar results were observed in each animal.

In an effort to ascertain if our *in vitro* data on BIRC3 phenotype had any *in vivo* relevance, we established subcutaneous xenograft tumor models of both control U251 and BIRC3 over-expression U251 HG cells. For all animals, we implanted the control U251 HG cells in the right flank while the BIRC3 over-expression U251 HG cells were implanted in the left flank. Animals were treated daily with oral TMZ at 50 mg/kg BW for 5 days after 2 weeks of tumor implantation and then subjected to non-invasive *in vivo* apoptosis imaging for detection of caspase-mediated apoptosis as measured by fluorescence. In response to TMZ treatment, we noted a significant resistance to caspase-mediated apoptosis in BIRC3 over-expression U251 HG cells compared to control U251 HG cells (Fig. 6a–c). This finding suggests that BIRC3 over-expression suppresses caspase activation in GBM. We further examined xenograft tissue by immunohistochemistry for caspase 3 staining. Immunohistochemistry results corroborated our *in vivo* apoptosis fluorescence imaging findings. We noted a marked decrease in caspase 3 activation and necrosis in BIRC3-overexpressing U251 GBM xenografts in response to TMZ treatment compared to wild-type U251 GBM xenografts (Fig. 7a–d). When wild-type U251 GBM xenografts were treated with TMZ, we observed large central areas of necrosis with marked increased in caspase 3 staining of cells adjacent to the necrosis/cell death zones (Fig. 7a,c). On the contrary, we did not observe any distinct regions of central necrosis with associated caspase activation, when BIRC3-overexpressing U251 GBM xenografts were treated with TMZ (Fig. 7b,d). These tissue IHC results confirm a potential role of BIRC3 expression in TMZ apoptosis resistance in GBM. However, when we examined tumor growth over our 5-day course treatment with TMZ, we did not observe any significant statistical difference in tumor size between control U251 GBM xenografts and BIRC3-overexpressing U251 GBM xenografts. It could relate to the fact that apoptosis and proliferation are distinct pathways and BIRC3 is predominantly within the apoptosis pathway. In addition, xenografts were sacrificed after the 5^th^ TMZ which is a short treatment course. Collectively, our *in-vivo* fluorescence and IHC data suggest that BIRC3 drives apoptosis evasion in GBM.

## Discussion

TMZ treatment and RT are the currently established standard of care paradigm for every newly diagnosed GBM patient pending no contraindications^1^. However, the combination of TMZ treatment and RT for GBM has significant limitations with respect to durable clinical response. Hence recurrence is always a rule for the disease. Most recently, several molecular predicators of favorable treatment response have been identified for high-grade gliomas. The most notable include 1p19q co-deletional status^38^, IDH1 mutation^38^ and O6-methylguanine-DNA methyltransferase (MGMT) promoter methylation status^42^. Tumors with 1p19q co-deletional status demonstrate favorable response to RT/TMZ and even for TMZ monotherapy. The absence of the MGMT promoter methylation in GBM is an established surrogate for TMZ resistance. In the absence of promoter methylation, MGMT enzyme transcription is uninterrupted thereby resulting in repair of TMZ-induced DNA alkylating events. Nonetheless, even patients with positive MGMT methylation status will invariably fail TMZ treatment, suggesting that there are potentially alternate mechanisms for TMZ drug resistance independent of methylation status.

In the present study, we focused on downstream apoptosis evasion substrates with special emphasis on IAPs. We have identified a novel mechanism of TMZ drug resistance resulting in apoptosis evasion in GBM. In particular, we identified BIRC3 - an IAP - as a robust prognosticating surrogate for TMZ and RT treatment resistance in GBM as noted with patient samples. Our work for the first time shows that TMZ and RT treatment induce BIRC3 gene expression up-regulation. This resulting up-regulation of BIRC3 confers onto human GBM cells as well as human GBM xenografts an apoptotic evasion phenotype. We further present new evidence that PI3K and STAT3 signaling contribute to up-regulation of BIRC3 gene up-regulation in GBM in response to RT. BIRC3 therefore emerges as a convergence point of apoptosis evasion in GBM mediated by the afore-mentioned pathways. More importantly, BIRC3 affords both prognostic as well therapeutic translational potential for GBM patients. Our findings therefore have several significant implications.

Primarily, the large TCGA data set provided an opportunity to fully assess the impact of several previously studied IAPs on the 5-year survival of GBM patients. BIRC3 was the only IAP that emerged as a statistically significant bona fide prognosticator in terms of differential expression. Patients with lower-folds expression of BIRC3 had a significantly improved overall 5-year survival compared to those with higher-fold expression. These findings were further validated using the GSE011 public dataset of 153 unique GBM samples with survival data. On the other hand, differential expression of Survivin (BIRC5) and XIAP (BIRC4) did not influence survival in GBM patients in the TCGA dataset. Survivin or XIAP resulted in an unfavorable outcome irrespective of differential expression folds. This is partly consistent with previous studies reporting expression of Survivin^11^ and XIAP^9^ in gliomas, which inversely correlated with an unfavorable outcome. However, the key implication of our findings is that BIRC3 is the only IAP whereby lower-folds expression could have a positive impact on long-term outcomes in GBM patients. Therefore, strategies that lead to down-regulation of BIRC3 expression or block BIRC3 activity could have a significant impact in the treatment of GBM in terms of durable response.

Secondly, the current standard therapy of TMZ treatment and RT for GBM results in up-regulation of BIRC3 as we have demonstrated herein. This phenomenon occurs almost immediately upon initiation of therapy, which suggests concurrent BIRC3-specific inhibition could be exploited for enhanced benefit. When GBM cells are rendered resistant to TMZ, there is marked up-regulation of BIRC3 expression. This is in agreement with a recent study, which showed that Survivin (BIRC5) expression was significantly increased in recurrent GBM compared with newly diagnosed tumors^43^. Similarly when GBM cells are genetically modified to over-express BIRC3, there is marked resistance to TMZ treatment thereby establishing an effect and causality relationship. This observation represents a novel alternate mechanism of TMZ drug resistance in GBM. With respect to radiotherapy, BIRC3 up-regulation effect was noted for even doses as lower as 2 Gy. Previous studies combining either an XIAP^39^ or a pan-IAP inhibitor^26^ with *in vitro* and *in vivo* irradiation resulted in enhanced sensitization to irradiation. Irradiation appears to modulate XIAP (BIRC4) protein levels through MDM2 expressions^44^. Hence in response to irradiation, MDM2 up-regulation results in translation increases in XIAP protein levels. Furthermore, there is recent evidence that XIAP is a potential stabilizer of BIRC3 in GBM^45^. Taken together, it would appear that RT treatment results in synergistic up-regulation and stabilization of BIRC3 expression. Our study uniquely characterizes the impact of TMZ and RT treatment on BIRC3 expression. Further studies are warranted to provide a better understanding of the temporal modulation of BIRC3 expression in response to TMZ and RT treatment. Such information could allow for optimized synergistic targeting of BIRC concurrent with the administration of TMZ and RT.

Thirdly, our studies have uncovered a new association between PI3K and STAT3 signaling with induction of BIRC3 up-regulation secondary to RT in GBM. Since PI3K and STAT3 are upstream to BIRC3, it is highly likely that apoptotic evasion signaling from either pathways converge on BIRC3. Furthermore, there is strong evidence in the literature that BIRC3 interacts with the tumor-necrosis factor alpha (TNF-alpha)/Nuclear Factor Kappa B (NFkB) signaling^46^,^47^,^48^,^49^,^50^. In GBMs, there is mechanistic evidence that the p65 component of NFkB transcriptionally contributes to up-regulation of BIRC3 expression in the presence of TNF-alpha ligand^50^. Hence BIRC3 appears to be a central point of therapeutic resistance in GBM. Interestingly, no studies to date have thoroughly examined mechanisms by which either PI3K or STAT3 pathways could result in BIRC3 up-regulation in GBM. We have demonstrated in the present study that blocking either STAT3 or PI3K pharmacological or genetically is sufficient to attenuate RT-induced up-regulation of BIRC3. We further demonstrate that PI3K effects are mediated mainly via its alpha-isoform and also that inhibition of this isoform is synergistic. The major implication here is that BIRC3 targeting could circumvent some of the current limitations with single-pathway targeting in GBM. Since BIRC3 is emerging as a convergence signaling point for apoptosis evasion, the limitations of pathway redundancies could be less of a problem. It is unclear if both PI3K and STAT3 pathways up-regulate BIRC3 expression independently or interdependently in GBM. However, there is recent evidence from proteomics analysis that PI3K and STAT3 pathways behave in interdependent manner in GBM^28^. Further studies are necessary to fully understand the molecular underpinnings of PI3K or STAT3 pathways signaling with respect to BIRC3. We have hereby established a new signaling node for BIRC3 in GBM as a convergence node for apoptosis evasion (Supplementary Figure 5).

Lastly, the BIRC3 phenotype results in a GBM that is refractory to highly cytotoxic TMZ and RT doses. In that regard, BIRC3 serves as a marker of aggressiveness in patient samples. More importantly it is a very translational target for synergy with cytotoxic GBM therapy. Smac mimetic non-specific inhibitors of BIRC proteins have been successfully employed in conjunction with cytotoxic strategies to promote therapeutic response in GBM cells^39^,^51^,^52^,^53^. Since therapeutic resistance remains the main major obstacle for GBM, our studies provide clinical rationale for designing small molecules as well as genetic strategies that selectively inhibit BIRC3.

## Additional Information

**How to cite this article**: Wang, D. *et al.* BIRC3 is a novel driver of therapeutic resistance in Glioblastoma. *Sci. Rep.* **6**, 21710; doi: 10.1038/srep21710 (2016).

## Supplementary Material

Supplementary Information
