# Supplementary material for: BIRC3 is a novel driver of therapeutic resistance in Glioblastoma
Source: Sci Rep. 2016 Feb 18;6:21710. doi: 10.1038/srep21710 (PMC4757860; doi:10.1038/srep21710)
Supplement: Supplementary Information [file srep21710-s1.pdf]

BIRC3 is a novel driver of therapeutic resistance in Glioblastoma

Dapeng Wang[1,2], Anders Berglund[3], Rajappa S. Kenchappa[1,2], Peter A. Forsyth[1,2], James J. Mulé[4,5], and Arnold B. Etame[1,2]

Author affiliation:

Departments of [1]Neuro-Oncology, [2]Tumor Biology, [3]Medical Bioinformatics, [4]Immunology, and [5]Cutaneous Oncology, Moffitt Cancer Center, 12902 Magnolia Drive, Tampa, FL 33612 USA

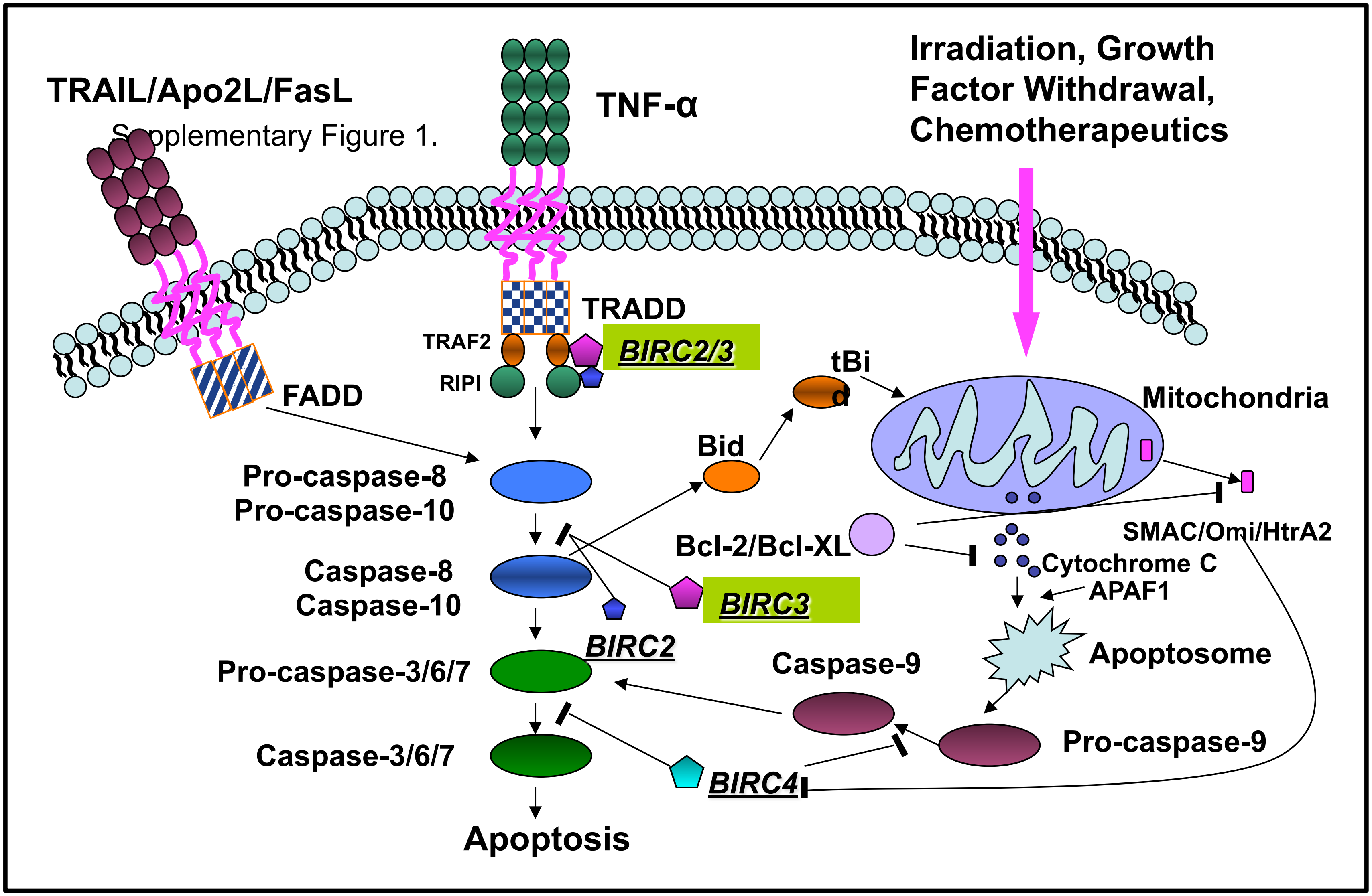

Supplementary Figure 1. BIRC family members regulate cell apoptosis.
Schematic representation of the role of BIRC3 in apoptotic evasion through their interactions with Caspases in the terminal phases of apoptosis. BIRC3 (green) inhibits activation of Caspase-8 and Caspase-10.

Supplementary Figure 2

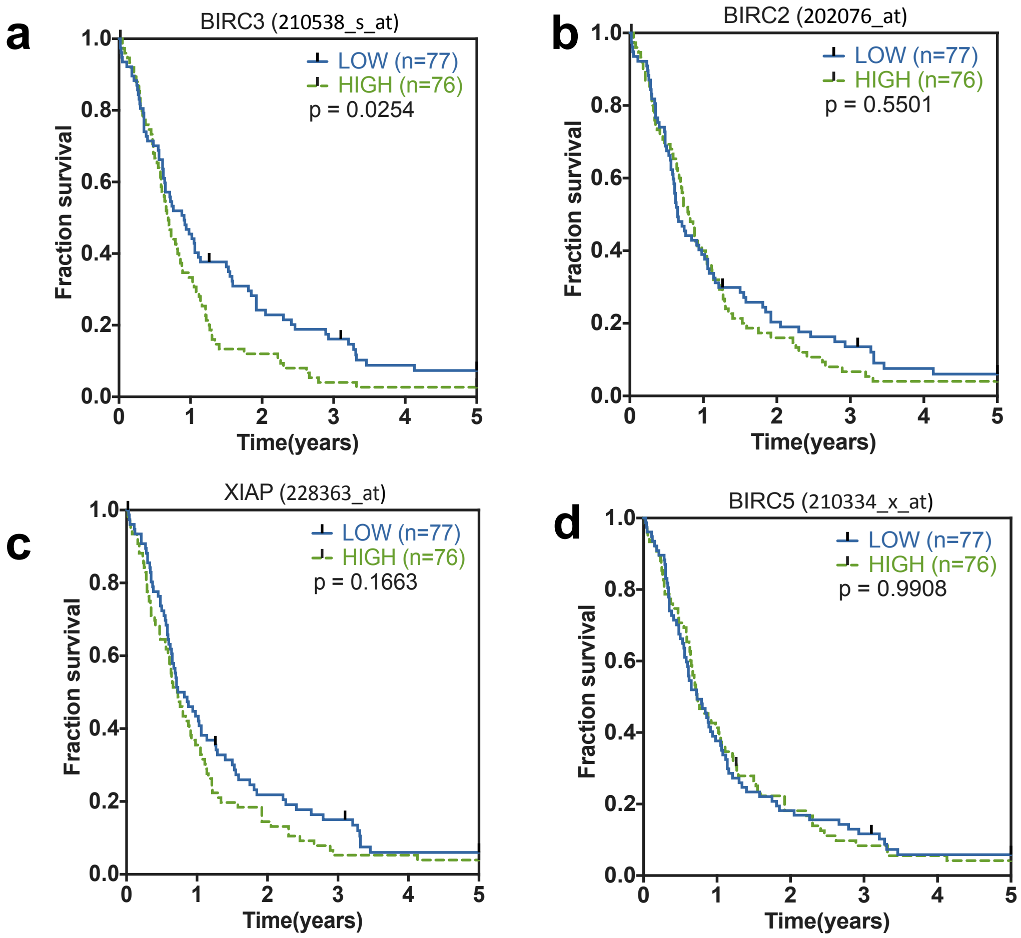

Supplementary Figure 2. GSE16011 showed BIRC3 expression was related with the survival of GBM patients.

The relationship of BIRC3, represented by two probesets, with GBM patients survival based on GSE011. 153 unique GBM samples on the Affymetrix U133 plus 2.0 chip were analyzed. Both probe sets for BIRC3(c-IAP2), 210538_s_at (a) and 230499_at(not shown) are related to 5-years overall survival in GSE011. BIRC2 (b), XIAP (c), and BIRC5 (d) are not related to survival.

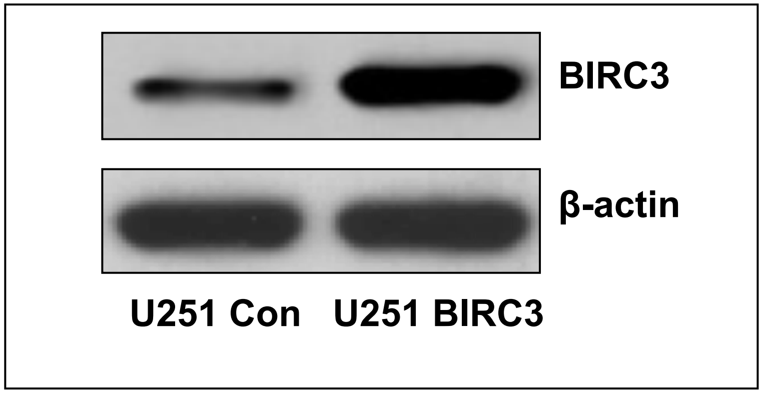

Supplementary Figure 3. BIRC3 is expressed at a higher level in U251 cells stably transfected with BIRC3.

The over expression of BIRC3 was verified by Western-blot for BIRC3 (see Materials and Methods for details). U251 control and U251 BIRC3 cells were cultured in T25 flasks and stimulated with 10ng/ml TNFα for 8 hours. Cells were harvested and BIRC3 expression was compared. One typical experiment from three independent experiments is shown here.

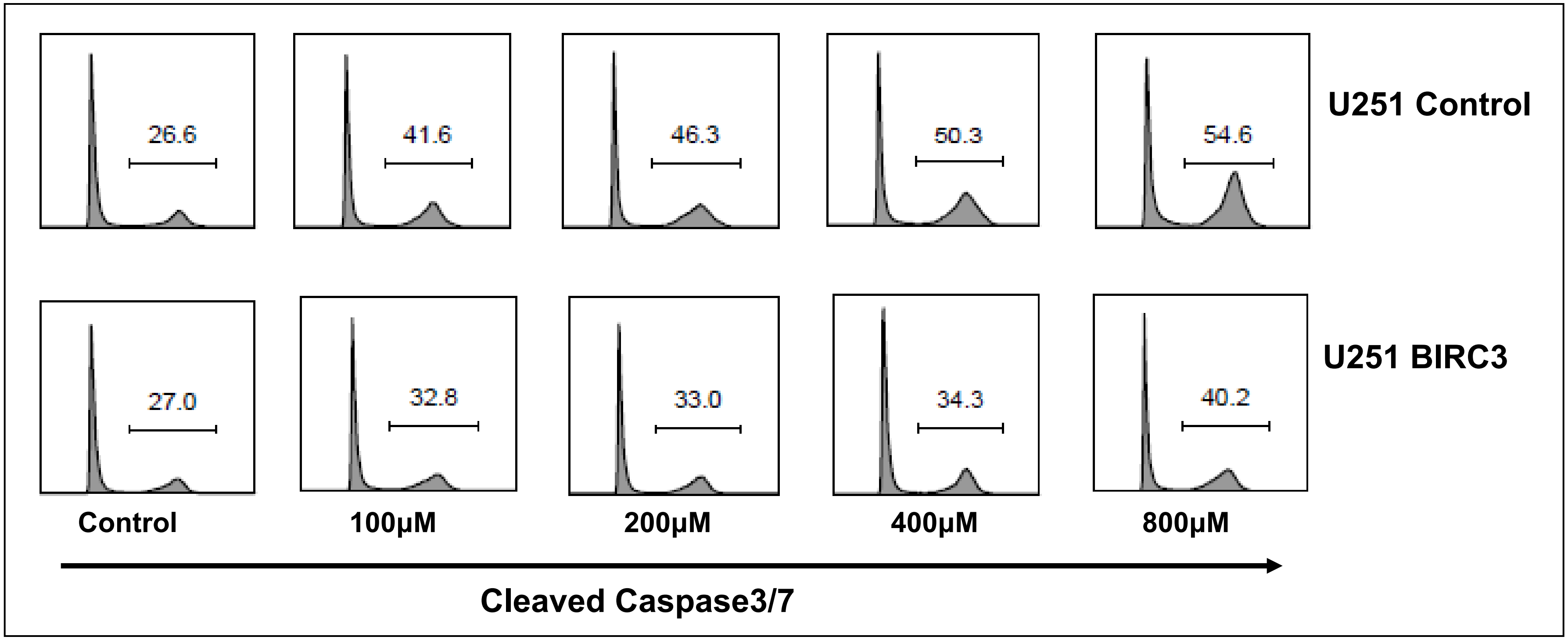

Supplementary Figure 4. Over-expression of BIRC3 leads to decreased activation of Caspase3/7 in U251 cells upon TMZ treatment.

1-2 × 10^4 U251 control or U251 BIRC3 cells were seeded in 24 well plates. Cells were then treated with 100 – 800 µM of TMZ for 5 days and harvested. 1 × 10^5 −1 × 10^7 cells were resuspended in 1 ml 0.1% BSA-PBS and stained with 1 µL of Cell Event Caspase-3/7 Green Detection Reagent; incubated for 25 minutes at 37°C. Samples were read on a BD LSRII flow cytometer and analyzed with FlowJo software (Tree Star, USA). The percentages of cleaved Caspase3/7 are shown. One typical experiment from three independent experiments is shown. ($p<0.05$; n=6).

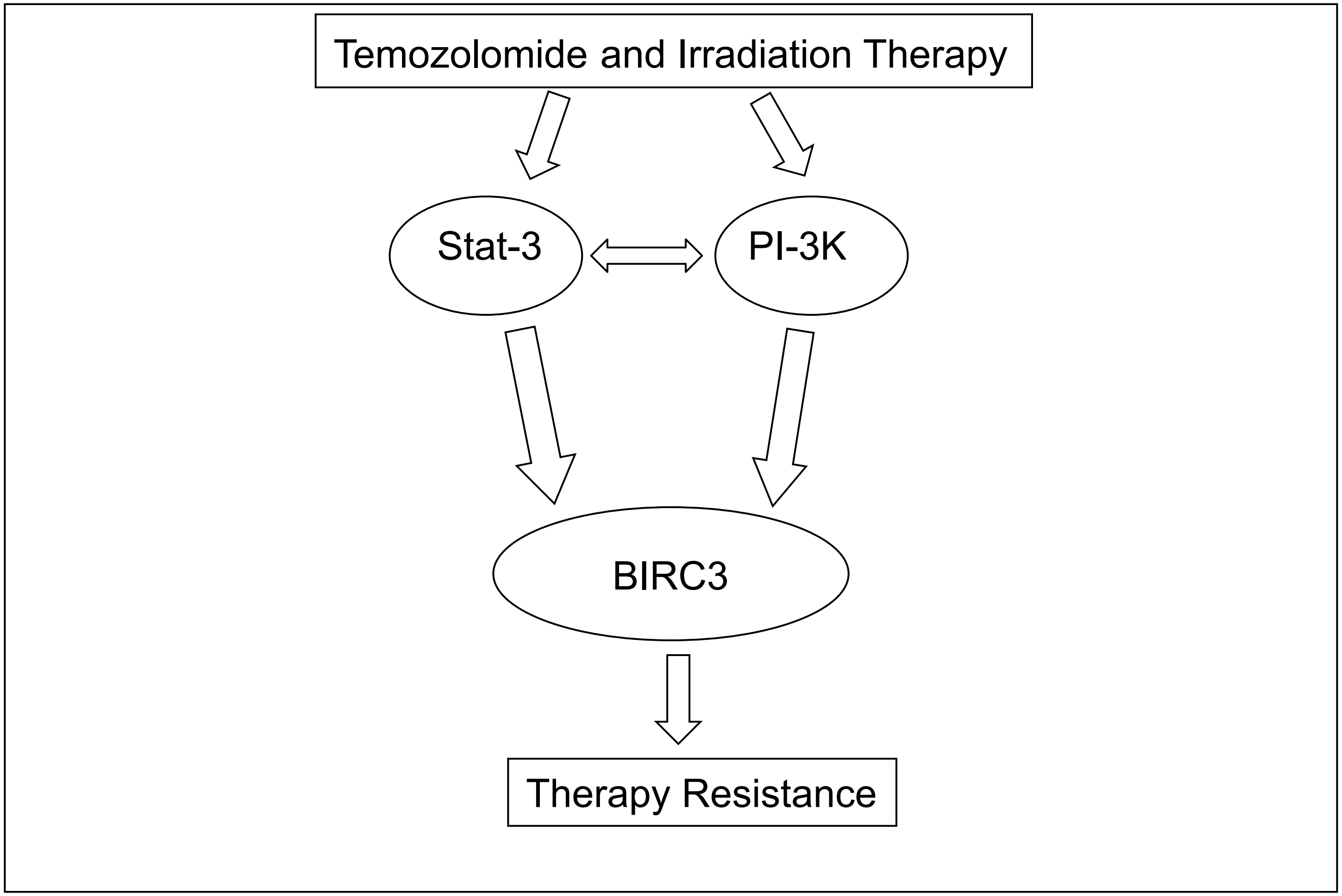

Supplementary Figure 5. Upregulation of BIRC3 Gene Expression in GBM following Radiotherapy and TMZ treatment.
Schematic representation of BIRC3 as a common signaling node for therapeutic resistance in GBM mediated through PI3K and STAT3 signaling cascades.
